# Hyperferritinemia and hypergammaglobulinemia predict the treatment response to standard therapy in autoimmune hepatitis

**DOI:** 10.1371/journal.pone.0179074

**Published:** 2017-06-08

**Authors:** Richard Taubert, Matthias Hardtke-Wolenski, Fatih Noyan, Claudine Lalanne, Danny Jonigk, Jerome Schlue, Till Krech, Ralf Lichtinghagen, Christine S. Falk, Verena Schlaphoff, Heike Bantel, Luigi Muratori, Michael P. Manns, Elmar Jaeckel

**Affiliations:** 1 Department of Gastroenterology, Hepatology and Endocrinology, Hannover Medical School, Hannover, Germany; 2 Department of Medical and Surgical Sciences, University of Bologna, Bologna, Italy; 3 Institute for Pathology, Hannover Medical School, Hannover, Germany; 4 Institute for Clinical Chemistry, Hannover Medical School, Hannover, Germany; 5 Institute of Transplantation Immunology and Integrated Research and Treatment Center Transplantation (IFB-Tx), Hannover Medical School, Hannover, Germany; Universita degli Studi di Pisa, ITALY

## Abstract

Autoimmune hepatitis (AIH) is a chronic hepatitis with an increasing incidence. The majority of patients require life-long immunosuppression and incomplete treatment response is associated with a disease progression. An abnormal iron homeostasis or hyperferritinemia is associated with worse outcome in other chronic liver diseases and after liver transplantation. We assessed the capacity of baseline parameters including the iron status to predict the treatment response upon standard therapy in 109 patients with untreated AIH type 1 (AIH-1) in a retrospective single center study. Thereby, a hyperferritinemia (> 2.09 times upper limit of normal; Odds ratio (OR) = 8.82; 95% confidence interval (CI): 2.25–34.52) and lower immunoglobulins (<1.89 times upper limit of normal; OR = 6.78; CI: 1.87–24.59) at baseline were independently associated with the achievement of complete biochemical remission upon standard therapy. The predictive value increased when both variables were combined to a single treatment response score, when the cohort was randomly split into a training (area under the curve (AUC) = 0.749; CI 0.635–0.863) and internal validation cohort (AUC = 0.741; CI 0.558–0.924). Patients with a low treatment response score (<1) had significantly higher cumulative remission rates in the training (p<0.001) and the validation cohort (p = 0.024). The baseline hyperferritinemia was accompanied by a high serum iron, elevated transferrin saturations and mild hepatic iron depositions in the majority of patients. However, the abnormal iron status was quickly reversible under therapy. Mechanistically, the iron parameters were not stringently related to a hepatocellular damage. Ferritin rather seems deregulated from the master regulator hepcidin, which was down regulated, potentially mediated by the elevated hepatocyte growth factor. In conclusion, baseline levels of serum ferritin and immunoglobulins, which are part of the diagnostic work-up of AIH, can be used to predict the treatment response upon standard therapy in AIH-1, although confirmation from larger multicenter studies is pending.

## Introduction

Autoimmune hepatitis (AIH) is an autoimmune disorder that affects all age groups and shows an increasing incidence [1]. Associations of genetic factors like MHC class II molecules [2] and external triggers like e.g. hepatitis A or Epstein-Barr virus infections [3] led to the hypothesis of an externally triggered break of tolerance in genetically predisposed individuals. Animal models, in which danger signals and genetic predispositions are both necessary to induce AIH, support this hypothesis [4, 5].

Once the diagnosis of an active AIH is made, an immunosuppressive medication that consists of predniso(lo)ne or alternatively budesonide in non-cirrhotic patients [6], with or without azathioprine is recommended [7]. Although complete biochemical remission (BR) can be achieved in about 80% of patients, a relapse after the withdrawal of immunosuppression is common, even in patients with a complete histological remission (CR) [8]. Furthermore, persistent inflammatory activity is associated with a histological disease progression and reduced survival [9–11]. As patients with incomplete biochemical response (IR) have worse prognosis a predictive identification of those patients could lead to a stronger immunosuppressive salvage therapy. Although, several studies described factors that are associated with worse outcome [12–14], none of those led to a practical risk stratification approach to predict the treatment response upon standard therapy.

An altered iron homeostasis mostly with hyperferritinemia, elevated transferrin saturation and/or an elevated serum iron is reported in multiple liver diseases beyond hemochromatosis and is mostly associated with worse disease course [15]. Additionally, markers of the intrahepatic iron homeostasis but not classical immune markers, predicted spontaneous operational tolerance after liver transplantation [16]. But so far there are no systematic reports about the iron homeostasis in AIH.

Thus, we assessed the predictive capacity of baseline parameters including the iron metabolism in AIH type 1 (AIH-1) to facilitate a risk stratification for the treatment response upon standard therapy.

## Material and methods

### Patients

We retrospectively included adult patients with untreated and biopsy proven AIH-1 between 1996 and 2015. Exclusion criteria were autoimmune overlap syndromes, replicative viral hepatitis, an AIH score <10 [17], a short term need for liver transplantation, bacterial infections or iron deficient anemia at diagnosis and no longitudinal follow up.

BR was defined as a persistent normalization of aminotransferases (alanine aminotransferase (ALT), aspartate aminotransferase (AST)) and immunoglobulin G (IgG) upon standard therapy with steroids (prednisolone or budesonide) and/or azathioprine [7]. IR was defined as improvement of these parameters without normalization under standard therapy over at least 2 years or a switch to a salvage therapy because of persistent disease activity. Patients that have been switched to a salvage therapy because of azathioprine intolerance were excluded. For the identification of predictors the cohort was randomly split into a training (2/3) and a validation cohort (1/3) (S1 Table). The comparator group of liver biopsies under therapy was matched to include various treatment responses and described recently [18]. In short, patients at biopsy were in mean 46.1±14.7 years old and in mean 4.4 ± 2.7 years under therapy.

This study was approved by the local research Ethics Committee of the Hannover Medical School. Written informed consent was obtained from each patient.

### Histology

In addition to the routine pathological review of the liver biopsies the iron content of the liver biopsies was assessed with the semi-quantitative total iron scoring system (range 0–60) described by Deugnier et al. [19] in a blinded fashion. This scoring system takes into consideration iron deposition into three compartments: hepatocytic, sinusoidal and portal.

Biopsies were processed for immunofluorescence as described previously [20] and mouse anti-human hepatocyte growth factor (HGF) antibody (Covalab, France) was applied.

### Detection of hepcidin-25 and cytokines in human sera

For quantitative measurement of hepcidin-25, the bioactive form of hepcidin, we used the Hepcidin ELISA (RE54061) according to the manufacturer’s instructions (IBL, Hamburg, Germany). Cytokine concentrations in patients sera were quantified by multiplex protein arrays, according to the manufacturer’s instruction (BioRad Laboratories, USA) as described [18]. In brief, a 2-laser array reader (Bio-Plex, BioRad Laboratories) simultaneously quantifies all cytokines of interest. Standard curves and concentrations were calculated with Bio-Plex Manager 4.1.1 on the basis of the 5-parameter logistic plot regression formula.

### Mice

Animal care and the induction of experimental murine autoimmune hepatitis (emAIH) were performed as reported previously [4]. Mice livers were analyzed at the peak of adenoviral hepatitis at day 21 after infection and 16 weeks after adenoviral infection for the assessment of untreated emAIH. Formalin fixed and paraffin embedded mice liver section were stained and scored for iron deposition similar to the human liver biopsies.

### Quantitative gene expression analysis

Isolation of RNA from formalin-fixed and paraffin-embedded human tissue, cDNA synthesis, preamplification of cDNA over 14 cycles and quantitative PCR analysis was performed as described previously [21]. PCR was run on a QuantStudio12K Flex Real-Time PCR System (Thermo Fisher Scientific, Germany). One 10μm section from representative liver biopsies with representative serum ferritin levels were selected from both treatment response groups (biochemical remission and incomplete biochemical response) according to availability of the remaining liver biopsy material after the routine clinical histopathological reviewing. Three reference genes (GAPDH, GUSB, POLR2A) were tested but GUSB and POLR2A were not reliably detectable in the pretrial or within this study. Thus expression of target genes could only be normalized to GAPDH. Gene expression was evaluated with the Expression Suite Software version 1.0.4 and Excel 2010. To quantify transcript levels, target gene Ct values were normalized using Ct values of GAPDH to generate–delta Ct values (-dCt).

### Statistical analysis

Statistical analysis was performed with SPSS 15.0 and GraphPad Prism 5. Mann-Whitney U tests were used for comparison of two and Kruskal-Wallis tests for comparisons of more than two groups. The Fisher’s exact test was used for contingency tables. Correlation analyses were calculated with the Spearman’s rank correlation. The Log rank test was used for the comparison of the cumulative treatment response rates. Area under the receiver operating characteristic (AUROC) analyses and the Youden’s index were used for the identification of cut-off values. P-values below 0.05 (two-tailed) were considered statistically significant in all analyses.

## Results

### Baseline parameters related to the subsequent treatment response in AIH-1

**Fig 1:**
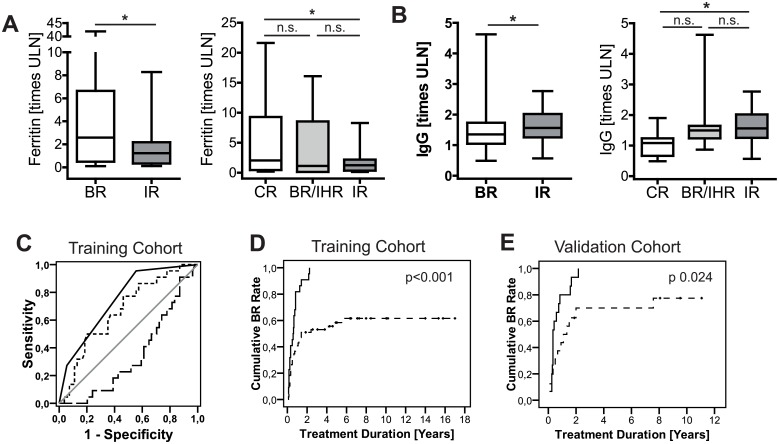
Hyperferritinemia and hypergammaglobulinemia were associated with the subsequent treatment response upon standard therapy in untreated AIH-1. (A) Left panel: Serum ferritin (SF) in untreated AIH-1 patients with subsequent biochemical remission (BR; N = 83) and incomplete biochemical response (IR; N = 26). Right panel: Histological treatment response with complete remission (CR: N = 16), BR with incomplete histological response (BR/IHR: N = 7) and IR (N = 26). (B) The same analysis for immunoglobulin G (IgG). (C) The AUROC analysis for the prediction of IR before the treatment initiation for SF (dashed line), IgG (dotted line) and their combined treatment response score (black solid line) in the retrospective trainings cohort (N = 76). (D) Rates of BR according to the treatment duration for the treatment response score<1 (solid line) and score≥1 (dashed line) for the training and (E) validation cohort. (* p<0.05; not significant, p≥0.05)

First baseline parameters of patients with untreated AIH-1 were compared according to their treatment response upon standard therapy in the total cohort of 109 patients (Table 1). Thereby, the fold changes of the upper limit of normal (ULN) were used for the majority of parameters to compensate for age and gender specific reference values. IgG, serum iron (SI) and serum ferritin (SF) were significantly different and AST showed a trend to be different between patients with subsequent BR and IR (Table 1). SI was excluded from further analyses because off redundant effects like SF but with lower fold changes between the two groups. In a multivariate binary logistic regression analysis SF (p = 0.002; OR = 8.82; 95% CI: 2.25–34.52) and IgG (p = 0.004; OR = 6.78; 95% CI: 1.87–24.59) but not AST (p = 0.199; OR = 2.97; 95% CI: 0.56–15.69) were independently associated with the treatment response (Fig 1A and 1B).

**Table 1:** Data of untreated AIH-1 patients according to subsequent treatment response upon standard therapy.

	Complete Responders		Incomplete Responders		p
	*Median (IQR)*	*n*	*Median (IQR)*	*n*	
Age at diagnosis (years)	54.7 (23.9)	83	50.0 (22.0)	26	0.235
Gender (male/female)	28 / 55		11 / 15		0.285
AIH score^a^	14.0 (4.0)	83	12.0 (4.0)	26	0.229
follow up time (months)	**53.9 (54.3)**	**83**	**81.3 (66.8)**	**26**	**0.005**
time to biochemical remission* (months)	6.5 (10.0)	78			
**Autoantibodies**					
ANA	72 / 83		21 / 26		0.321
SMA	61 / 82		18 / 24		0.590
SLA	5 / 76		1 / 22		1.000
pANCA	21 / 24		9 / 10		0.666
**Laboratory test**					
IgG (times ULN)	**1.35 (0.69)**	**83**	**1.56 (0.77)**	**26**	**0.034**
Alanine aminotransferase (times ULN)	21.3 (27.3)	83	16.4 (21.9)	26	0.184
Aspartate aminotransferase (times ULN)	20.3 (28.7)	83	18.3 (19.0)	25	0.054
Glutamate dehydrogenase (times ULN)	5.0 (5.0)	59	3.8 (3.0)	21	0.118
Gamma-glutamyl transferase (times ULN)	4.4 (6.3)	83	4.8 (5.9)	24	0.638
Alkaline phosphatase (times ULN)	1.3 (1.0)	82	1.4 (0.8)	26	0.301
Bilirubin (times ULN)	4.3 (13.6)	80	3.1 (9.8)	26	0.368
Prothrombin time (%)	70.0 (30.0)	82	76.5 (34.5)	22	0.243
Albumin (g/l)	35.0 (8.0)	67	35.5 (7.8)	18	0.690
***Iron homeostasis***					
Hemoglobin (g/dl)	13.5 (1.9)	83	13.2 (1.5)	26	0.127
Serum iron (μmol/l)	**1.26 (0.79)**	**67**	**0.81 (0.45)**	**19**	**0.003**
Transferrinsaturation (%)	50.5 (48.0)	58	35.0 (23.0)	17	0.081
Iron binding capacity of transferrin (μmol/l)	60.0 (24.0)	60	58.0 (15.0)	17	0.645
Ferritin (times ULN)	**2.59 (6.16)**	**83**	**1.23 (1.85)**	**26**	**0.035**
Soluble transferrin receptor (nM)	14.5 (6.8)	18	13.8 (12.2)	5	0.881
Ferritinindex (sTfR / logFerritin)	0.40 (0.21)	17	0.45 (0.81)	5	0.649
Hepcidin (ng/ml)	1.8 (4.3)	21	5.2 (6.8)	8	0.114
***Acute phase proteins***					
C-reactive protein (mg/l)	7.5 (12.0)	80	7.0 (8.0)	23	0.921
TNF alpha (ng/ml)	23.4 (32.7)	53	35.8 (20.2)	14	0.194
IL6 (ng/ml)	19.1 (19.5)	52	21.0 (55.9)	14	0.461
**Histology**					
mHAI	9.0 (4.0)	58	9.0 (3.0)	14	0.976
Ishak A	3.0 (1.0)	58	3.5 (1.0)	14	0.684
Ishak B	0.0 (2.0)	58	0.0 (1.0)	14	0.638
Ishak C	2.0 (1.0)	58	2.0 (2.0)	14	0.739
Ishak D	3.0 (1.0)	58	3.0 (2.0)	14	0.410
Fibrosis	3.0 (4.0)	63	3.0 (4.0)	17	0.643
Hepatic iron score**					
total	0.0 (4.0)	47	0.0 (3.0)	10	0.598
hepatocytic	0.0 (0.0)	47	0.0 (0.0)	10	0.444
sinusoidal	0.0 (1.0)	47	0.0 (0.0)	10	0.217
portal	0.0 (0.0)	47	0.0 (0.0)	10	0.426
**Induktion therapy**					
Prednisolone/Budesonide	74 / 5		20 / 5		0.058
Prednisolone dose (mg/day)	60.0 (10.0)	74	60.0 (10.0)	20	0.952
Budesonide dose (mg/day)	9.0 (-)	5	9.0 (-)	5	1.000
Azathioprine	52 / 83		16 / 10		0.548
Azathioprine dose (mg/day)	50.0 (50.0)	27	100.0 (62.5)	9	0.079

^a^ according to Alvarez et al. [17];

*documented at our center according to AASLD guidelines 2010 [7];

** according to Deugnier et al. 1993 [19].

When the histological treatment response was included, patients with CR had significantly higher SF and lower IgG levels than patients with IR. Patient with BR and incomplete histological remission (BR/IHR) had intermediate levels (Fig 1A and 1B).

Due to the retrospective analysis, there was no standardized immunosuppression regimen. However, we compared at least the induction therapy that was available in 95.4%. The missing patients were included in a randomized, double blinded trail to compare budesonide with prednisone both combined with azathioprine [6]. There was no significant difference in the initial administration of steroids and azathioprine, while there was a trend to more budesonide usage in patients subsequent IR. Additionally, there was no significant differences in the drug doses between BR and IR of patients that actually received prednisolone, budesonide or azathioprine (Table 1).

To test whether the different baseline parameters could predict the subsequent treatment response, we first contacted several large volume centers for external validation cohorts, but the numbers of patients with available clinical data about the iron status or the baseline biomaterial to measure the iron status was underpowered for a statistical analysis. Although the University of Bologna had a well characterized large cohort as recently published [13], only 17 patients had biomaterial for the iron status paired to the treatment endpoints of the present study.

Thus we divided our cohort randomly into a training and internal validation cohort (S1 Table). Both IgG and SF were significantly associated with the treatment response upon standard therapy in an AUROC analysis in the training cohort. Due to the small sample number the association was confirmed with a binary logistic regression analysis in the training cohort (Table 2). However, the area under the curve (AUC) was higher, when both parameters were combined to one treatment response score (sum of: ferritin: <2.09x ULN = 1 point; >2.09x ULN = 0 points; IgG: >1.89x ULN = 1 point; <1.89x ULN = 0 points) (Fig 1C, Table 3). Additionally, patients with a treatment response score <1 had significantly higher cumulative biochemical remission rates than those with a score ≥1 (Fig 1D). This combined score performed with comparable results in our internal validation cohort (Fig 1E, Table 3). In summary, the treatment response score enriched those patients with subsequent IR upon standard therapy (Table 4) and was inversely correlated with the serological but not with the histological severity of hepatitis (S2 Table).

**Table 2:** AUROC and univariate analysis for the prediction of incomplete biochemical remission upon standard therapy in untreated AIH-1 in the training cohort.

	AUROC		Binary logistic regression		
	AUC	Confidence Interval	Cut-off	Odds Ratio	Confidence Interval
**Ferritin**	0.656	0.531–0.782	< 2.09x ULN	3.88	1.31–11.47
**IgG**	0.668	0.539–0.797	> 1.89x ULN	3.91	1.35–11.35

**Table 3:** Diagnostic performance of the treatment response score to predict incomplete treatment response in untreated AIH-1.

Cohort	Training	Validation
**AUC**	0.749	0.741
**Confidence Interval**	0.635–0.863	0.558–0.924
**Sensitivity (Cut off 1 / 2)**	0.96 / 0.27	1.00 / 0.0
**Specificity (Cut off 1 / 2)**	0.44 / 0.94	0.52 / 0.97
**Positive predictive value (Cut off 1 / 2)**	0.41 / 0.67	0.22 / 0.00
**Negative predictive value (Cut off 1 / 2)**	0.96 / 0.76	1.00 / 0.88

**Table 4:** Enrichment for incomplete biochemical remission upon standard therapy with an increasing treatment response score in the total cohort.

		Incomplete Responders	Complete Responders
**Treatment response score**	0	1 (2.5%)	39 (97.5%)
	1	19 (32.2%)	40 (67.8%)
	2	6 (60.0%)	4 (40.0%)
	Total	26 (23.9%)	83 (76.1%)

### Reversible hyperferritinemia and mild iron deposition in untreated AIH-1

**Fig 2:**
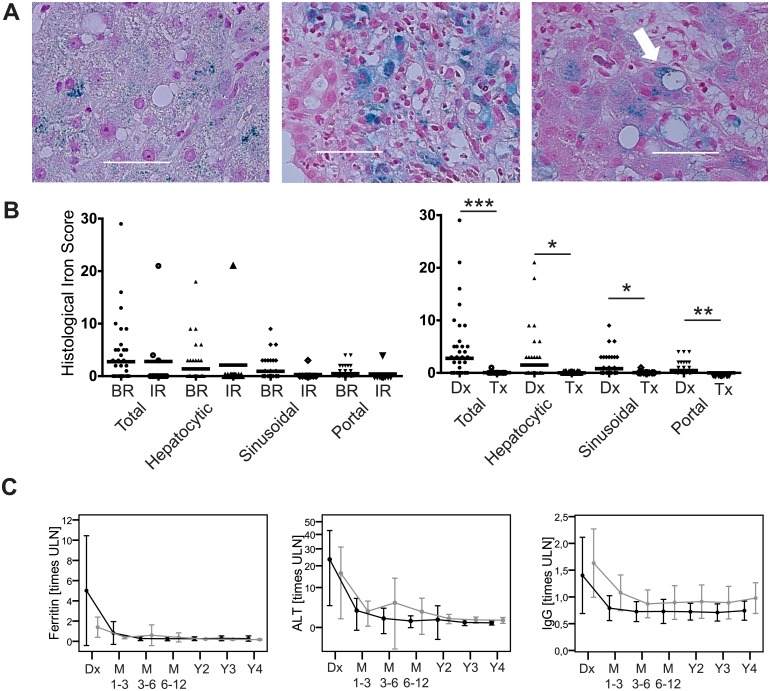
Reversible hyperferritinemia and mild iron deposition in untreated AIH-1. (A) Intrahepatic iron deposition (blue granula) in untreated AIH-1 in (left) hepatocytes, (middle) portal fields and (right) the sinusoidal compartment (white arrow). (B) The semi-quantitative histopathological iron deposition score (left) in untreated AIH-1 with subsequent biochemical remission (BR: N = 47) or incomplete response (IR: N = 10) and (right) under therapy (Tx; N = 24) compared to baseline at diagnosis (Dx; N = 61). (C) Longitudinal course (mean and standard deviation) under therapy (M = month; Y = year) in BR (black) and IR (grey). (* p<0.05; ** p<0.01; *** p<0.001; ULN = upper limit of normal).

Since an additional iron overload is associated with worse outcomes in other liver diseases [15], we further explored the iron parameters in the total cohort and found elevations in the majority of the 109 patients with untreated AIH-1 (hyperferritinemia in 65%, elevated SI in 58% and elevated transferrin saturation in 48% of patients) (S1 Table). Iron was mildly deposed in the liver irrespective of the subsequent treatment response (Table 1, Fig 2A and 2B). However, the hyperferritinemia and the iron deposition normalized under therapy (Fig 2B and 2C).

Mice with an experimental murine AIH (emAIH) showed an increased iron deposition after 16 weeks but not during the acute, self-limited adenoviral hepatitis at 3 weeks that is used to break tolerance and is characterized by much higher aminotransferase levels than in the chronic emAIH phase (S1 Fig) [4].

### Mechanisms of altered iron homeostasis

**Fig 3:**
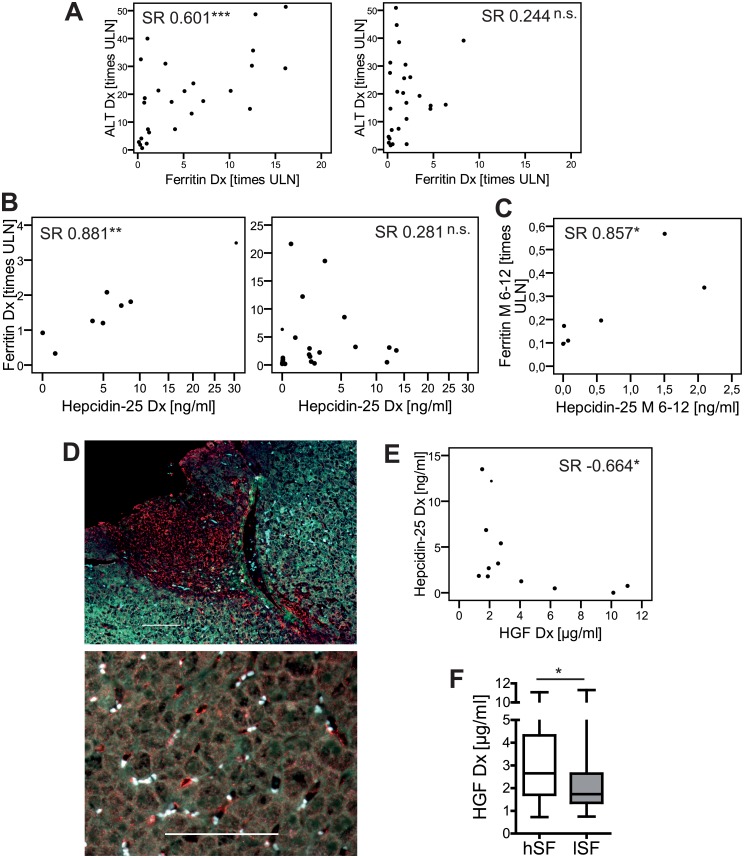
Iron homeostasis is potentially deregulated by HGF driven suppression of hepcidin-25 in untreated AIH-1. (A) Spearman rank correlation (SR) analysis of serum ferritin in AIH-1 with alanine aminotransferase (ALT) at diagnosis (Dx) in patients with subsequent biochemical remission (BR, left panel, N = 24) and incomplete biochemical response (IR, right panel, N = 24) matched for ALT, gender and age as far as possible. (B) SR analysis of serum ferritin and hepcidin-25 in patients with IR (N = 8; left panel) and with BR (N = 21; right panel) upon standard therapy and (C) in patients with achieved BR after 6–12 months of therapy (M 6–12; right; N = 7). (D) The hepatocyte growth factor (HGF, red; autofluorescence in green and blue) is expressed in (top) the portal tracts, endothelium and (bottom) liver sinusoids in a representative liver biopsy of untreated AIH-1. White bars represent 100 μm. (E) SR analysis of HGF and hepcidin-25 in untreated AIH-1 (N = 12) with subsequent BR and high ferritin (>2,09x ULN). (F) HGF in patients with high (hSF, N = 30) and low serum ferritin (lSF, N = 37). (* p<0.05; ** p<0.01; *** p<0.001; not significant, p≥0.05; ULN = upper limit of normal)

Next we were interested in the etiology of the differential SF and SI levels. Baseline SF correlated with aminotransferase levels in patients with BR but not with IR (S3 Table). Although this could point to a release from damaged hepatocytes [22], the higher levels of SF, SI and also the lower levels of IgG persisted in BR, when patients were matched for ALT, gender and age as far as possible (Fig 3A, S4 Table). As an acute phase reactant SF was correlated with C-reactive protein (CRP) and IL6 (SR = 0.266; p = 0.031; n = 66) but not with IL1β and TNFα (data not shown).

Hepcidin-25, the bioactive master regulator of the iron homeostasis, was reduced (median < 5 percent percentile of normal ((13.3 ng/ml) according to manufacturer’s protocol) in untreated AIH-1 (Table 1, S1 Table). In healthy individuals SF is correlated with hepcidin-25 [16]. However, such a correlation was only found at baseline in patients with subsequent IR but not with BR (Fig 3B). Nonetheless, this correlation was regained after 6–12 months of standard therapy in patients achieving BR (Fig 3C). Unfortunately, follow-up samples of IR patients were not available. Beside this Hepcidin-25 was not significantly correlated with any other baseline laboratory parameter of liver inflammation or iron homeostasis (ALT, AST, AP, bilirubin, mHAI, CRP, prothrombin time, SI, transferrin saturation) in a Spearman rank correlation analysis of the total cohort or in patients with subsequent BR or IR.

Mouse in-vitro data showed a suppression of the hepcidin transcription by the hepatocyte growth factor (HGF) via the bone morphogenetic protein pathway (BMP) [23]. Similar to animal models, HGF was mostly expressed in the portal tracts, endothelial layers and liver sinusoids in untreated human AIH-1 (Fig 3D). Interestingly, we found an inverse correlation of serum HGF levels and hepcidin-25 at baseline in patients with BR and high SF (>2.09x ULN) (Fig 3E) as well as higher HGF levels in patients with high SF compared to those with low SF (cut-off 2.09x ULN) (Fig 3F).

In order to further explore the molecular regulation, we analyzed the expression of 18 genes that are associated with the iron homeostasis in 12 formalin-fixed and paraffin-embedded liver biopsies. Unfortunately, only ferritin heavy chain 1 (FTH1), BMP receptor 1A (BMPR1A) and hemojuvelin (HFE2) could be reliably detected. However, the intrahepatic baseline gene expression of FTH1, BMPR1A and HFE2 was not different between BR and IR (S3 Fig) thereby offering no stringent explanation for the differentially regulated iron homeostasis.

## Discussion

Multiple factors have been associated with the disease course in AIH. Predictors of mortality and the need for transplantation are e.g. prolonged time to BR, AIH type 2 and anti-SLA positivity. Furthermore anti-SLA was associated with an increased disease activity in other studies but not with the subsequent treatment response in the current study. The prognostic relevance of cirrhosis was ambiguous in numerous studies [12, 13, 24]. To our knowledge, SF is the first predictor of subsequent BR upon standard therapy in untreated AIH-1. Although other studies had linked higher IgG levels to worse outcome or higher relapse rates after cessation of therapy [14, 25, 26], an AUROC analysis for the prediction of IR was not published so far. The combination of both values, SF and IgG, complements each other and facilitates a more precise prediction. So far the predictive capacity is not strong enough to base a clinical decision just on these two laboratory values. However, the score can contribute additional information to the clinical decision process, especially in the current absence of better prognostic markers. The score is designed to identify primarily those patients with subsequent IR that need more medical surveillance. Thus patients with null score points seem to have an excellent prognosis to reach BR (98%) upon standard therapy, even when their inflammatory activity is high (Table 1 and S2 Table). Although patients with one and two score point are enriched for IR (36%) this is not sufficient to impact the initial induction therapy. But when BR is not reached e.g. within 12 or 24 months the chance of these patients not to reach BR increase to 64% and 81% (Fig 1D and 1E). Hopefully this score can be further improved by additional parameters in future study from larger multicenter initiatives.

In parallel to this report our collaborators found age and cirrhosis at baseline to be predictive for the treatment response upon standard therapy in a large retrospective cohort [13]. However, differences to the present study were the missing analysis of iron parameters in the majority of patients and milder disease presentations with lower treatment responses rates in Bologna. Moreover, they applied looser criteria for the definition of IR. While we demanded at least a treatment duration of 24 months, an arbitrary threshold to not misclassify patients that reached BR later, their minimum follow up in the IR cohort was 3 months. However a treatment duration of at least 24 months was recently agreed upon a meeting of the International Autoimmune Hepatitis Group (IAIHG). Additionally, the follow-up in the Hannover cohort was about twice as long as in Bologna (Table 1). Transplant centers like the Hannover Medical School are probably biased to more severe AIH manifestations and adequate cut-offs will only be generated by larger multi center initiatives. Nonetheless, several clinical reports support our finding that a more severe initial presentation of AIH results in a favorable long-term prognosis [27–29].

Interestingly none of the histological parameters were associated with the treatment response in our study, while baseline and cumulative inflammation were risk factors for a fibrosis progression in a Scandinavian cohort. Additionally cumulative IgG load was associated with a fibrosis progression [30]. This underlines the current recommendations to achieve BR to prevent disease progression. A risk stratification for the treatment response already at the time point of diagnosis with the two clinical routine blood parameters SF and IgG, which can be determined inexpensively, hopefully facilitates an earlier switch to a salvage therapy as discussed above.

We were surprised about the association of the iron parameters with the treatment response. Based on this first systematic assessment of the iron parameters in AIH, a hyperferritinemia with a mild intrahepatic iron deposition, both quickly reversible under therapy, seems to be common in untreated AIH-1. In contrast a persistent hyperferritinemia after liver transplantation was associated with a higher mortality [31, 32]. It is interesting to note that the development of operational tolerance after liver transplantation was also related to markers of the intrahepatic iron homeostasis [16]. The lack of detrimental sequela of an iron overload in AIH-1, as it is found in other chronic liver disease, may be due to its short duration and reversibility under therapy.

Regarding mechanistic aspects we found no stringent evidence for a mere release of SF and SI from damaged hepatocytes [22] as an explanation for the differences between BR and IR, when patients were matched aminotransferase levels. However, there seems to be an association with the disease severity (ALT, CRP, IL6) in terms of acute phase reactions at least in those with BR. In contrast to other inflammatory diseases, which are characterized by a hyperferritinemia with elevated hepcidin but without an iron overload (reduced transferrin saturation and SI), e.g. as in lupus erythematosus and rheumatoid arthritis [33–35], the hyperferritinemia in AIH-1 with subsequent BR appears to be deregulated from hepcidin. At least the reduced hepcidin levels have recently been confirmed on the transcriptional and protein level [36]. In summary, our results in untreated AIH-1 are compatible with a model of a HGF driven repression of hepcidin (Fig 3F) as demonstrated in mouse studies [23]. Since HGF has pleiotropic effects regarding immunomodulation, regeneration and tissue repair this may also be relevant to the different treatment responses in AIH [37]. In accordance with the results in humans, elevated iron storages were also seen in subsequent chronic emAIH, but not in the more severe acute adenoviral hepatitis that is necessary to break the liver tolerance.

Chronic liver diseases beyond hemochromatosis, like non-alcoholic fatty liver disease (NAFLD), chronic hepatitis C (CHC) and alcoholic liver diseases (ALD), can also be associated with an iron overload [15, 38, 39]. A relevant distinction towards the etiology of this iron overload is again the hepcidin level, which is elevated in NAFLD and decreased in CHC and ALD. In contrast to untreated AIH-1, the hepcidin down-regulation seems to be in part virus related in CHC and associated with hepatocellular oxidative stress and TNFα release from stress activated Kupffer cells in ALD [39].

In summary, hyperferritinemia and hypergammaglobulinemia at diagnosis can predict the treatment response upon standard therapy. Thereby, patients with low ferritin and high IgG, who do not reach BR within e.g. the first 12 months, might be candidates for an early switch to a second line therapy or those with high SF and low IgG might be candidates for an early immunosuppression minimization, although exact cut-off values of both baseline parameters need to be determined from ongoing and larger multi center consortia.

## Supporting information

S1 Table Data of untreated AIH-1 patients. (DOC)

S2 Table Correlation analysis of the treatment response score with liver inflammation. (DOC)

S3 Table Correlation analysis of ferritin and regulators of the iron homeostasis in untreated AIH-1. (DOC)

S4 Table Data of matched untreated AIH-1 patients according to subsequent treatment response upon standard therapy. (DOC)

S1 Fig Intrahepatic iron deposition in experimental murine AIH. The iron deposition score in murine livers with adenoviral hepatitis without features of AIH (n = 8) 3 weeks and untreated experimental murine autoimmune hepatitis (emAIH, n = 10) 16 weeks after the adenoviral infections for the induction of emAIH. Horizontal bars represent the median. (** p<0.01)
(PDF)

S2 Fig Intrahepatic expression of genes related to the iron homeostasis in untreated AIH-1. (A) Serum ferritin levels paired to the formalin-fixed and paraffin-embedded liver biopsies of untreated AIH-1 of whom gene expression analysis was performed. The subsequent treatment response upon standard therapy is indicated as biochemical remission (BR, blue dots) or incomplete biochemical response (IR, red triangles). (B) Relative expression as -delta-Ct (-dCt = GAPDH–target gene: higher–dCt values corresponds to higher gene expression) of the genes that could be detected repetitively. All comparisons of the gene expression between BR and IR were not significant. Horizontal bars represent the median. (C) Correlation analysis with the Spearman rank correlation coefficient (SR) of the intrahepatic ferritin heavy chain 1 (FTH1) with serum ferritin levels.
(PDF)
