# Supplementary material for: Hyperferritinemia and hypergammaglobulinemia predict the treatment response to standard therapy in autoimmune hepatitis
Source: PLoS One. 2017 Jun 8;12(6):e0179074. doi: 10.1371/journal.pone.0179074 (PMC5464635; doi:10.1371/journal.pone.0179074)
Supplement: S1 Table — (DOC) [file pone.0179074.s001.doc]

**S1 Table.** Data of untreated AIH-1 patients.

|  | **Overall** | | | | **Training cohort** | | | | **Validation cohort** | | | |
| --- | --- | --- | --- | --- | --- | --- | --- | --- | --- | --- | --- | --- |
|  | *Median (IQR)* | *n* | | | *Median (IQR)* | *n* | | | *Median (IQR)* | *n* | | |
| Age at diagnosis (years) | 52.2 (24.1) | 109 | | | 49.3 (27.8) | 76 | | | 56.6 (17.8) | 33 | | |
| Gender (male/female) | 39 / 70 |  | | | 27 / 49 |  | | | 12 / 21 |  | | |
| AIH score^a^ | 14.0 (4.0) | 109 | | | 13.5 (4.0) | 76 | | | 14.0 (4.0) | 33 | | |
| follow up time (months) | 57.6 (61.5) | 109 | | | 58.3 (61.8) | 76 | | | 52.9 (62.4) | 33 | | |
| **Autoantibodies** |  |  | | |  |  | | |  |  | | |
| ANA^b^ | 93 / 109 |  | | | 62 / 76 |  | | | 31 / 33 |  | | |
| SMA^b^ | 79 / 106 |  | | | 55 / 76 |  | | | 24 / 30 |  | | |
| SLA | 6 / 104 |  | | | 4 / 73 |  | | | 2 / 31 |  | | |
| pANCA | 30 /34 |  | | | 20 / 21 |  | | | 10 / 13 |  | | |
| **Laboratory test** |  |  | | |  |  | | |  |  | | |
| IgG (times ULN) | 1.41 (0.77) | 109 | | | 1.48 (0.85) | 76 | | | 1.36 (0.57) | 33 | | |
| Alanine aminotransferase (times ULN) | 19.3 (25.2) | 108 | | | 20.7 (22.7) | 75 | | | 16.1 (22.7) | 33 | | |
| Aspartate aminotransferase (times ULN) | 20.3 (25.7) | 109 | | | 21.2 (29.0) | 76 | | | 18.3 (25.8) | 33 | | |
| Alkaline phosphatase (times ULN) | 1.4 (0.9) | 108 | | | 1.4 (1.0) | 75 | | | 1.2 (0.7) | 33 | | |
| Gamma-glutamyl transferase (times ULN) | 4.5 (6.0) | 107 | | | 4.5 (6.3) | 75 | | | 4.3 (6.2) | 32 | | |
| Bilirubin (times ULN) | 3.6 (13.3) | 106 | | | 4.0 (12.9) | 73 | | | 1.7 (14.8) | 33 | | |
| Prothrombin time (%) | 70.0 (31.0) | 104 | | | 69.0 (32.5) | 73 | | | 74.0 (30.0) | 31 | | |
| Albumin (g/l) | 35.0 (7.0) | | 85 | 35.0 (5.8) | | | 61 | 35.0 (9.5) | | | 24 |  |
| ***Iron homeostasis*** |  |  | | |  |  | | |  |  | | |
| Hb (d/dl) | 13.4 (1.8) | 109 | | | 13.2 (1.6) | 76 | | | 14.3 (1.5) | 33 | | |
| Serum iron (µmol/l) | 1.10 (0.70) | 86 | | | 1.12 (0.73) | 61 | | | 1.04 (0.67) | 25 | | |
| Transferrinsaturation (%) | 44.0 (45.0) | 75 | | | 49.0 (46.0) | 53 | | | 37.5 (43.0) | 22 | | |
| Iron binding capacity of transferrin (µmol/l) | 59.0 (22.0) | 77 | | | 58.0 (23.0) | 55 | | | 61.5 (25.0) | 22 | | |
| Ferritin (times ULN) | 2.07 (5.4) | 109 | | | 2.08 (4.9) | 76 | | | 2.05 (6.5) | 33 | | |
| Soluble transferrin receptor (nM) | 13.8 (5.7) | 23 | | | 14.1 (4.7) | 16 | | | 11.6 (8.4) | 7 | | |
| ***Acute phase reactant*** |  |  | | |  |  | | |  |  | | |
| C-reactive protein (mg/l) | 7.0 (10.0) | 103 | | | 7.0 (12.0) | 72 | | | 8.0 (9.0) | 31 | | |
| **Histology** |  |  | | |  |  | | |  |  | | |
| mHAI | 9.0 (4.0) | 71 | | | 9.0 (3.0) | 47 | | | 8.0 (6.0) | 24 | | |
| Fibrosis (Ishak) | 3.0 (4.0) | 80 | | | 3.0 (4.0) | 63 | | | 2.0 (4.0) | 27 | | |

^a^ according to Alvarez et al.[17]; ^b^ according to AASLD guidelines 2010 [7].
