# Supplementary material for: Hyperferritinemia and hypergammaglobulinemia predict the treatment response to standard therapy in autoimmune hepatitis
Source: PLoS One. 2017 Jun 8;12(6):e0179074. doi: 10.1371/journal.pone.0179074 (PMC5464635; doi:10.1371/journal.pone.0179074)
Supplement: S2 Table — (DOC) [file pone.0179074.s002.doc]

**S2 Table. Correlation analysis of the treatment response score with** liver inflammation.

|  | SR | p | N |
| --- | --- | --- | --- |
| **Laboratory test** |  |  |  |
| Alanine aminotransferase (times ULN) | -0.413 | <0.001 | 109 |
| Aspartate aminotransferase (times ULN) | -0.433 | <0.001 | 108 |
| Alkaline phosphatase (times ULN) | -0.149 | n.s. | 108 |
| Bilirubin (times ULN) | -0.407 | <0.001 | 106 |
| **Histology** |  |  |  |
| mHAI | -0.132 | n.s. | 71 |

SR=Spearman rank correlation coefficient; N=sample number; n.s.=no significant correlation
