# Supplementary material for: Hyperferritinemia and hypergammaglobulinemia predict the treatment response to standard therapy in autoimmune hepatitis
Source: PLoS One. 2017 Jun 8;12(6):e0179074. doi: 10.1371/journal.pone.0179074 (PMC5464635; doi:10.1371/journal.pone.0179074)
Supplement: S3 Table — (DOC) [file pone.0179074.s003.doc]

**S3 Table.** Correlation analysis of ferritin and regulators of the iron homeostasis in untreated AIH-1.

|  |  | overall | | | BR | | | IR | | |
| --- | --- | --- | --- | --- | --- | --- | --- | --- | --- | --- |
|  |  | SR | p | N | SR | p | N | SR | p | N |
| Ferritin | ALT | 0.558 | <0.001 | 109 | 0.603 | <0.001 | 80 | n.s. | | 26 |
| Ferritin | CRP | 0.512 | <0.001 | 103 | 0.568 | <0.001 | 80 | n.s. | | 26 |
| Ferritin | mHAI | n.s. | | 71 | n.s. | | 58 | n.s. | | 26 |
| Ferritin | IgG | n.s. | | 109 | n.s. | | 83 | n.s. | | 26 |

BR=patients with subsequent biochemical remission; IR=patients with subsequent incomplete biochemical response; SR=Spearman rank correlation coefficient; N=sample number; n.s.=no significant correlation.
