# Supplementary material for: Hyperferritinemia and hypergammaglobulinemia predict the treatment response to standard therapy in autoimmune hepatitis
Source: PLoS One. 2017 Jun 8;12(6):e0179074. doi: 10.1371/journal.pone.0179074 (PMC5464635; doi:10.1371/journal.pone.0179074)
Supplement: S4 Table — (DOC) [file pone.0179074.s004.doc]

**S4 Table.** Data of matched untreated AIH-1 patients according to subsequent treatment response upon standard therapy.

|  | **Complete Responders** | | **Incomplete Responders** | | **p** |
| --- | --- | --- | --- | --- | --- |
|  | *Median (IQR)* | *n* | *Median (IQR)* | *n* |
| Age at diagnosis (years) | 54.4 (20.7) | 26 | 50.0 (22.0) | 26 | 0.213 |
| Gender (male/female) | 11 / 15 |  | 10 / 16 |  | 1.000 |
| **Laboratory test** |  |  |  |  |  |
| IgG (times ULN) | **1.24 (0.58)** | **26** | **1.56 (0.77)** | **26** | **0.027** |
| Alanine aminotransferase (times ULN) | 18.1 (23.3) | 26 | 16.4 (21.9) | 26 | 0.728 |
| Aspartate aminotransferase (times ULN) | 19.2 (29.5) | 26 | 18.3 (19.0) | 25 | 0.318 |
| Glutamate dehydrogenase (times ULN) | 4.4 (4.0) | 18 | 3.8 (3.0) | 21 | 0.460 |
| Gamma-glutamyl transferase (times ULN) | 4.5 (6.3) | 26 | 4.8 (5.9) | 24 | 0.627 |
| Alkaline phosphatase (times ULN) | 1.3 (1.0) | 26 | 1.4 (0.8) | 26 | 0.319 |
| Bilirubin (times ULN) | 5.3 (10.0) | 26 | 3.1 (9.8) | 26 | 0.475 |
| Prothrombin time (%) | 78.0 (33.5) | 25 | 76.5 (34.5) | 22 | 0.966 |
| ***Iron homeostasis*** |  |  |  |  |  |
| Hb (g/dl) | 13.3 (1.8) | 26 | 13.2 (1.5) | 26 | 0.370 |
| Serum iron (µmol/l) | **1.36 (0.59)** | **19** | **0.81 (0.45)** | **19** | **0.001** |
| Transferrinsaturation (%) | **60.0 (59.0)** | **17** | **35.0 (23.0)** | **17** | **0.013** |
| Iron binding capacity of transferrin (µmol/l) | 55.0 (27.0) | 18 | 58.0 (15.0) | 17 | 0.909 |
| Ferritin (times ULN) | **3.35 (9.90)** | **26** | **1.23 (1.85)** | **26** | **0.034** |
| ***Acute phase proteins*** |  |  |  |  |  |
| CRP (mg/l) | 8.0 (13.0) | 25 | 7.0 (8.0) | 23 | 0.992 |
| **Histology** |  |  |  |  |  |
| mHAI | 8.0 (4.0) | 17 | 9.0 (3.0) | 13 | 0.432 |
| Fibrosis | 2.0 (4.0) | 19 | 3.0 (4.0) | 17 | 0.471 |

Patients were matched for ALT, gender and as far as possible for age at diagnosis.
