# Supplementary figures and images for: Hyperferritinemia and hypergammaglobulinemia predict the treatment response to standard therapy in autoimmune hepatitis
Source: PLoS One. 2017 Jun 8;12(6):e0179074. doi: 10.1371/journal.pone.0179074 (PMC5464635; doi:10.1371/journal.pone.0179074)

S1 Figure

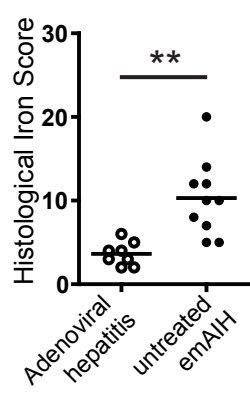

Supplement: S1 Fig — The iron deposition score in murine livers with adenoviral hepatitis without features of AIH (n = 8) 3 weeks and untreated experimental murine autoimmune hepatitis (emAIH, n = 10) 16 weeks after the adenoviral infections for the induction of emAIH. Horizontal bars represent the median. (** p<0.01) (PDF) [file pone.0179074.s005.pdf]

S2 Figure

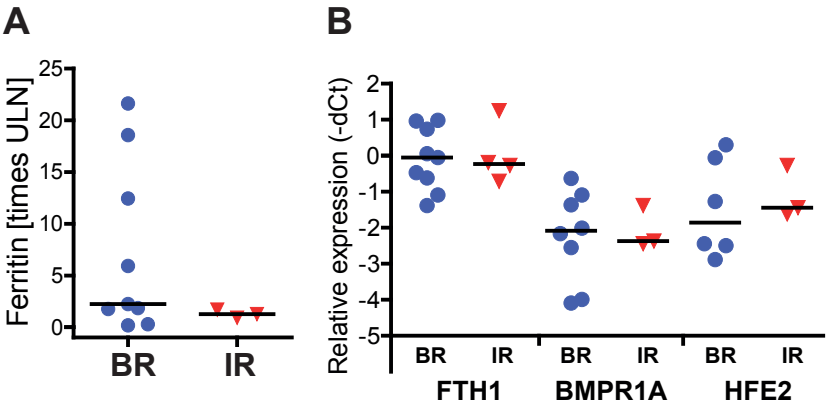

Supplement: S2 Fig — (A) Serum ferritin levels paired to the formalin-fixed and paraffin-embedded liver biopsies of untreated AIH-1 of whom gene expression analysis was performed. The subsequent treatment response upon standard therapy is indicated as biochemical remission (BR, blue dots) or incomplete biochemical response (IR, red triangles). (B) Relative expression as -delta-Ct (-dCt = GAPDH–target gene: higher–dCt values corresponds to higher gene expression) of the genes that could be detected repetitively. All comparisons of the gene expression between BR and IR were not significant. Horizontal bars represent the median. (C) Correlation analysis with the Spearman rank correlation coefficient (SR) of the intrahepatic ferritin heavy chain 1 (FTH1) with serum ferritin levels. (PDF) [file pone.0179074.s006.pdf]
